# Advances in Understanding Mitochondrial MicroRNAs (mitomiRs) on the Pathogenesis of Triple-Negative Breast Cancer (TNBC)

**DOI:** 10.1155/2021/5517777

**Published:** 2021-03-22

**Authors:** Hung-Yu Lin, Pei-Yi Chu

**Affiliations:** ^1^Research Assistant Center, Show Chwan Memorial Hospital, Changhua 500, Taiwan; ^2^Department of Pathology, Show Chwan Memorial Hospital, Changhua 500, Taiwan; ^3^School of Medicine, College of Medicine, Fu Jen Catholic University, New Taipei City 242, Taiwan; ^4^Department of Health Food, Chung Chou University of Science and Technology, Changhua 510, Taiwan; ^5^National Institute of Cancer Research, National Health Research Institutes, Tainan 704, Taiwan

## Abstract

Triple-negative breast cancer (TNBC) is characterized by poor outcome and the most challenging breast cancer type to treat worldwide. TNBC manifests distinct profile of mitochondrial functions, which dictates reprogrammed metabolism, fosters tumor progression, and notably serves as therapeutic targets. Mitochondrial microRNAs (mitomiRs) are a group of microRNAs that critically modulate mitochondrial homeostasis. By a pathway-centric manner, mitomiRs tightly orchestrate metabolic reprogramming, redox status, cell apoptosis, mitochondrial dynamics, mitophagy, mitochondrial DNA (mtDNA) maintenance, and calcium balance, leading to an emerging field of study in various cancer types, including TNBC. We herein review the recent insights into the roles and mechanism of mitomiRs in TNBC and highlight its clinical value in diagnosis and prognosis as well as vital advances on therapeutics of preclinical and clinical studies.

## 1. Introduction

Breast cancer (BC) is the most common cancer in women globally, accounting for about a quarter of female cancer [1, 2]. In spite of recent improvements in molecular and imaging diagnosis and treatments including hormone therapy, target therapy, chemotherapy, and radiotherapy, BC remains the leading cause of cancer death worldwide [1–3]. Based on the expression of estrogen receptor (ER), progesterone receptor (PR), human epidermal growth factor 2 (HER2), and proliferation marker protein Ki-67, BC can be categorized into four subtypes including luminal A, luminal B, HER2, and triple-negative breast cancer (TNBC) [4].

**Figure 1:**
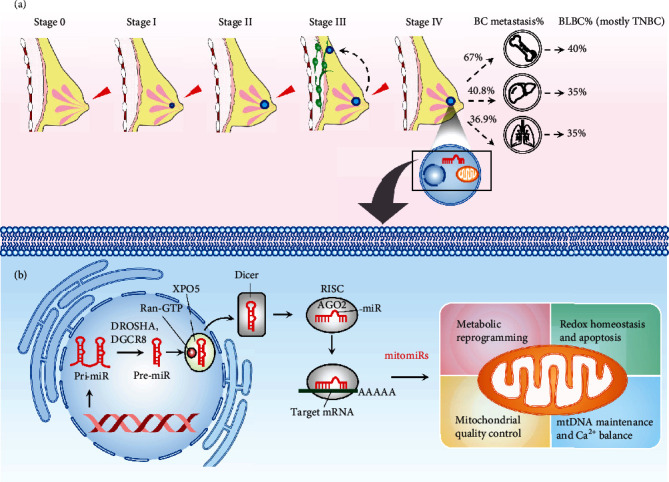
Graphic overview depicting the roles of mitomiRs in modulating mitochondrial homeostasis and its involvements in the scenario of TNBC progression. (a) TNBC stages classified by TNM system [7]. Advanced breast cancer comprises inoperable locally advanced breast cancer and metastatic (stage IV) breast cancer. The bone, the liver, and the lungs account, respectively, for about 67%, 40.8%, and 36.9% of the common metastatic sites, wherein basal-like BC (BLBC, accounting for 75% of the TNBC subtypes [6]) hits 40%, 35%, and 35%, respectively, of the metastatic BC [7]. (b) The mitomiR biogenesis and its impacts on mitochondrial dimensions. The precursor transcripts (pri-miR) are transcribed and posttranscriptionally cleaved by microprocessor (DROSHA and DGCR8) in the nucleus to liberate the pre-miR hairpin. The pre-miR is then exported to the cytoplasm by exportin 5 (XPO5) bound to guanosine 5′-triphosphate (Ran-GTP). In the cytoplasm, the DICER endoribonuclease cleaves the loop of the pre-miR to produce the mature miR. The resulting miR embeds in a groove of Argonaute (AGO) of the RNA-induced silencing complex (RISC) and ultimately targets a strand of mRNA by base pairing its 3′ untranslated region (UTR). When bound to an mRNA, RISC inhibits translation, yet the main effect is to degrade the mRNA through deadenylation. mitomiRs are a particular cluster of miRs that predominantly occupy a sphere of influence on dimensions of mitochondrial homeostasis, including metabolic reprogramming, redox homeostasis, mitochondrial quality control, mtDNA maintenance, and Ca^2+^ balance.

TNBC is tested negative for ER, PR, and HER2 by immunohistochemical staining, accounting for 15-20% of BC cases [5]. Advanced breast cancer comprises inoperable locally advanced breast cancer and metastatic (stage IV) breast cancer. As shown in Figure 1(a), the bone, the liver, and the lungs account, respectively, for about 67%, 40.8%, and 36.9% of the common metastatic sites, wherein basal-like BC (BLBC, accounting for 75% of the TNBC subtypes [6]) hits 40%, 35%, and 35%, respectively, of the metastatic BC [7]. More than 70% of the metastatic TNBC cases fails to survive after five years of diagnosis and exhibits worse prognosis than other BC subtypes [8]. Due to the lack of ER/PR/HER2 receptors, TNBC is unresponsive to endocrine and target therapy, such as tamoxifen, aromatase inhibitors, and/or anti-HER2-targeted therapies. As such, surgery, radiotherapy, and predominantly nonspecific chemotherapy (e.g., anthracycline and taxane regimens) remain the mainstay for management of these patients, yet with severe side effects worsening quality of life [9, 10]. Although the combination uses of chemotherapy regimens (e.g., capecitabine in conjunction with taxanes) have shown increased response rates, the multiagent approach led to increased toxicities [11, 12]. Therefore, treatments for TNBC have since a major challenge for oncologists due to the absence of unambiguous molecular targets.

Mitochondria are dynamic organelles that produce energy through oxidative phosphorylation (OXPHOS) for the sophisticated biochemical reactions of a cell. Originated from eubacterial endosymbiosis, each of them has multiple copies of mitochondrial DNA (mtDNA), which is a circular, double-stranded DNA (16,569-base pairs in human) encoding 37 genes, including 13 proteins, 22 tRNAs, and 2 rRNAs [13]. Structurally, mitochondria have double-membrane system, composed of outer membrane and inner membrane, which compartmentalize intermembrane space, crista, and matrix where OXPHOS takes place to generate ATP [13]. More than intracellular powerhouses, mitochondria play a central role in controlling physiological process and cellular fate by orchestrating metabolism, redox status, apoptosis, dynamics, mitophagy (a mechanism selectively degrading mitochondria by autophagy), mtDNA maintenance, and calcium balance [14, 15]. More recently, approaches aiming at disturbances of cellular metabolism and mitochondrial functions for the treatment of cancer, including BC, have been emerging [15, 16].

MicroRNAs (miRs) are small, approximately 22-nucleotide noncoding RNAs. miR genes are derived from within the introns and are subject to similar types of epigenetic regulation as are the protein-coding genes [17]. As shown in Figure 1(b), a miR gene is initially transcribed by RNA polymerase II into a primary miRNA (pri-miRNA) and then processed in the nucleus by microprocessor complex comprising an endoribonuclease Drosha and a double-stranded RNA-binding protein DiGeorge syndrome critical region 8 (DGCR8) into a ~70 nt precursor miR (pre-miR) [18], which are then exported to the cytoplasm by an exportin 5/guanosine 5′-triphosphate (XPO5/RanGTP) complex. In the cytoplasm, the loop of a pre-miRNA is cleaved by the endonuclease Dicer to generate a mature miR [19]. The regulatory functions of a mature miR are accomplished by associating with the Argonaute (AGO) of the RNA-induced silencing complex (RISC) [20]. As such, a miR ultimately targets a strand of mRNA by base pairing its 3′ untranslated region (UTR) and negatively regulates mRNA expression in most cases. When bound to an mRNA, RISC inhibits translation, yet the main effect is to degrade the mRNA through deadenylation [17].

mitomiRs are a group of miRs that intimately regulate mitochondrial functions. mitomiRs can exert the repressive effect on gene expression to modulate mitochondrial functions by two ways: one major type of mitomiRs targets mRNAs in the cytoplasm; the other type of mitomiRs is imported into mitochondria to target mtDNA-encoded mRNAs. With regard to the gene origin of the mitomiRs, the vast majority of them are nuclear-encoded, while few of the mitomiRs are encoded by mtDNA (e.g., miR-1974, miR-1977, and miR-1978) [21].

Mounting evidence in recent years has noted the translational implication of mitomiRs in metabolic disorder, degenerative diseases, and cancer [22–24]. Their functions in modulating multiple aspects of mitochondrial homeostasis permit alterations in cancer metabolism, growth, metastasis, and sensitivity to clinical drugs. More importantly, mitomiRs' potential as a predictive panel and therapeutic targets is notably emerging. As such, we herein focus on the novel biological roles and mechanisms of mitomiRs on the mitochondria-centric metabolic network in the context of TNBC (Figure 1). Moreover, we highlight recent insights into the advanced understating of predictive value and therapeutic implication of mitomiRs.

## 2. Roles of mitomiRs in Regulating Metabolic Reprogramming of TNBC

Cancer cells are characterized by significant metabolic reprogramming which facilitate survival and rapid proliferation in a nutrient-deprived tumor microenvironment [25, 26]. Otto Warburg firstly identified that cancer cells maintain high levels of glycolysis for ATP production, regardless of oxygen availability, a phenomenon thereafter termed as the Warburg effect [27]. Since then, an increasing volume of study focused on cancer-associated metabolic reprogramming within crucial metabolic pathways, including altered metabolism of glucose, fatty acid, and amino acids as well as perturbed OXPHOS, in an attempt to find out metabolic susceptibilities during cancer progression. The potential preclinical/clinical reagents targeting metabolic reprogramming of TNBC have been reviewed (32296646). In this regard, we summarized a number of mitomiRs that have been reported to significantly regulate TNBC progression by acting to metabolic reprogramming (Table 1).

Several lines of study noted that mitomiRs perturb glycolysis-associated targets to inhibit TNBC. Jiang et al. have demonstrated that miR-155/miR-143 axis modulates tumor development by dictating glycolysis [40]. They showed that miR-155 and miR-143 have TNBC-promoting and TNBC-inhibiting effect, respectively. Mechanistically, tumorigenic inflammatory signals augment glycolysis through miR-155, which suppresses miR-143 by targeting CCAAT-enhancer-binding protein *β* (C/EBP*β*). The resulting decrease in miR-143 leads to an unleashed targeting inhibition on hexokinase 2 (HK2), leading to an increase in HK2 and consequently an induced glycolysis [40]. miR-18a was demonstrated to inhibit TNBC growth and metastasis in both *in vivo* and *in vitro* models and expression of hypoxic genes [39]. The molecular basis of miR-18a lies in the direct binding to the *HIF1A* UTR [39]. Inhibition of TNBC proliferation *in vitro* can be achieved by the overexpression of miR-101, which exerts repressive effect on gene expression of AMPK [38]. Yu et al. have shown that miR-340 inhibits glycolysis through modulating mitochondrial calcium homeostasis in TNBC [37]. Specifically, miR-340 targets the UTR of mitochondrial calcium uniporter (MCU) gene and represses the expression to reduce tumor growth and metastasis [37]. Li et al. demonstrated that miR-30a inhibits glycolysis and enhances mitochondrial respiratory activity by targeting lactate dehydrogenase A (LDHA), whereby the growth and metastasis of TNBC is reduced [35]. miR-342 was reported to disturb glucose metabolism and lactate uptake of TNBC by targeting monocarboxylate transporter 1 (MCT1) [34]. miR-128 was shown to specifically inhibit insulin receptor (INSR) and insulin receptor substrate 1 (IRS1) to abate glycolytic signaling pathway and mitochondrial respiration, resulting in decreased tumor growth [33]. Both miR-148a and miR-152 were shown to directly target pyruvate kinase M1/2 (PKM2) to counteract glycolytic metabolism of TNBC cells, leading to decreased cancer cell proliferation and angiogenesis [46]. Yao et al. have identified that let-7a represses signal transducers and activators of transcription 3 (STAT3) expression level to subsequently mediate hnRNPA1/PKM2 signaling, leading to a decrease in glucose metabolism and TNBC proliferation [31]. miR-140 showed antiglycolytic and antiproliferative effect on TNBC through direct binding to glucose transporter 1 (GLUT1) [30]. Li et al. showed that miR34a inhibitor promotes cell proliferation and glucose uptake, indicating the anti-TNBC effect of miR34a [47]. More recently, Wang et al. demonstrated that miR-29a overexpression exerts inhibitory effect on proliferation, migration, and invasion of TNBC cells through targeting the anterior gradient 2 (ARG2)/HIF-1*α* axis [28]. Furthermore, circular RNA circPVT1 was identified as a miR sponge, leading to suppressing the expression of miR-29a and promoting *in vivo* tumor growth [28].

In addition to glucose metabolism, some mitomiRs act toward fatty acid metabolism to exert inhibitory effect on TNBC. For example, miR-195 was shown to target acetyl-CoA carboxylase (ACACA), fatty acid synthase (FASN), and 3-hydroxy-3-methylglutaryl CoA reductase (HMGCR) to suppress epithelial-mesenchymal transition (EMT), proliferation, migration, and invasion [43]. Serguienko et al. have demonstrated that let-7a targets SCD (stearoyl-CoA desaturase) to reduce cell proliferation and sensitivity to doxorubicin [42]. More recently, Chen et al. reported that miR-1291 targets estrogen-related receptor alpha (ERR*α*) to inhibit the expression level of carnitine palmitoyltransferase 1C (CPT1C), causing a decrease in cell proliferation *in vitro* and tumor growth *in vivo* [41].

With regard to amino acid metabolism, Ueda et al. demonstrated that miR-27a play a suppressive role over TNBC by disturbing glutamine/cystine dynamics. Specifically, miR-27a directly bind to UTR of cystine/glutamate transporter (xCT) and cystathionine gamma-lyase (CTH), whereby cystine uptake and production, respectively, are compromised, leading to a decrease in mammosphere formation and cancer stem cell markers as well as an increase in sensitivity to doxorubicin and paclitaxel [36]. On the other hand, some mitomiRs were shown to curb TNBC by impeding OXPHOS. miR-4485 and miR-663 have been identified to hinder mitochondrial function by direct binding to the UTR of mtDNA-encoded 16S rRNA and ubiquinol-cytochrome c reductase complex assembly factor 2 (UQCC2), respectively [44, 45].

**Figure 2:**
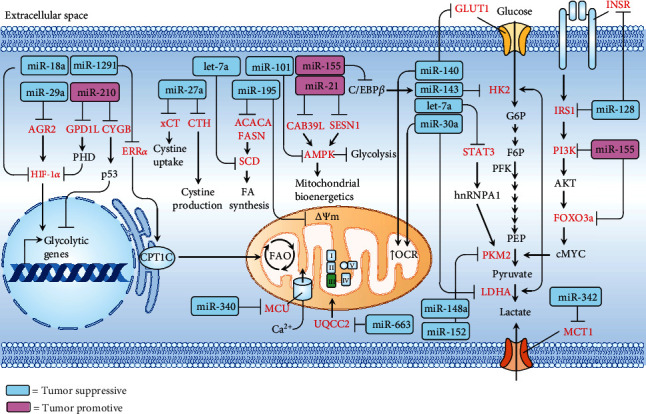
Integrative network depicting that tumor-suppressive (blue rectangle) or tumor-promotive (pink rectangle) mitomiRs act on specific target genes (red) that are involved in metabolic reprogramming. *ΔΨ*m: mitochondrial membrane potential; ACACA: acetyl-CoA carboxylase; AGR2: anterior gradient 2; AMPK: AMP-activated protein kinase; CAB39L: calcium-binding protein 39-like; C/EBP*β*: CCAAT-enhancer-binding protein *β*; CPT1C: carnitine palmitoyltransferase 1C; CTH: cystathionine gamma-lyase; CYGB: cytoglobin; ERR*α*: estrogen-related receptor alpha; F6P: fructose 6-phosphate; FASN: fatty acid synthase; G6P: glucose 6-phosphate; GLUT1: glucose transporter 1; GPD1L: glycerol-3-phosphate dehydrogenase 1; HIF-1*α*: hypoxia-inducible factor 1-alpha; HK2: hexokinase 2; INSR: insulin receptor; IRS1: insulin receptor substrate 1; LDHA: lactate dehydrogenase A; MCT1: monocarboxylate transporter 1; MCU: mitochondrial calcium uniporter; mtDNA: mitochondrial DNA; mt-ROS: mitochondrial reactive oxygen species; OCR: oxygen consumption rate; PEP: phosphoenolpyruvate; PFK: phosphofructokinase; PI3K: phosphoinositide 3-kinases; PKM2: pyruvate kinase M1/2; SCD: stearoyl-CoA desaturase; SESN1: sestrin-1; STAT3: signal transducers and activators of transcription 3; UQCC2: ubiquinol-cytochrome c reductase complex assembly factor 2; xCT: cystine/glutamate transporter.

In contrast, some reports have explored that a group of mitomiRs responsible for metabolic reprogramming can act to promote the development of TNBC. In this regard, miR-21 was shown to exert its function by targeting calcium-binding protein 39-like (CAB39L) and sestrin-1 (SESN1) to promote proliferation and invasion of TNBC [48]. Kim et al. have reported that miR-155 promotes glycolysis and growth of TNBC by targeting an axis comprising phosphoinositide 3-kinase regulatory subunit 1, forkhead box O, and c-MYC (PI3R1-FOXO3a-cMYC axis) [32]. A recent report conducted by Du et al. identified that miR-210 exerts a positive effect in glucose metabolism, lactate production, extracellular acidification rate (ECAR), proliferation, and starvation-induced apoptosis. The molecular underpinnings were found to lie in direct binding of miR-210 to GPD1L and CYGB, whereby HIF-1*α* stabilization and p53 suppression can, respectively, be maintained [29]. An integrative network summarizing the roles and pathways of mitomiRs is illustrated in Figure 2.

## 3. Roles of mitomiRs in Redox Homeostasis and Cell Apoptosis

The increased demand for cellular ATP and accelerated oxidation of nutrient molecules trigger the generation of reactive oxygen species (ROS) derived from electron transport chain of mitochondria [49]. A moderate elevated ROS benefits cellular proliferation, whereas excessive ROS production can lead to oxidative damage to proteins, lipids, and DNA [50]. As such, intracellular ROS status plays a critical role in cell growth and survival. Indeed, a cluster of miRs functions to dictate the imbalance between the production of ROS and the activity of antioxidant defence system, resulting in the disruption of redox homeostasis and ultimately cancer cell apoptosis [22]. A study conducted by Eades et al. has demonstrated that miR-200a serves as repressor on TNBC cells by perturbing antioxidant defence mechanism [51]. Specifically, miR-200a directly targets Kelch-like ECH-associated protein 1 (KEAP1), leading to the NF-E2-related factor 2 (NRF2) stabilization and nuclear translocation to act forward the activation of Nrf2-dependent NAD(P)H-quinone oxidoreductase 1 (NQO1) gene transcription [51]. By targeting SCD, let-7a is able to alter fatty acid metabolism, leading to boosted mitochondrial activity and oxidative stress, as evidenced by an increase in oxygen consumption rate (OCR), ROS expression level, and the expression level of superoxide dismutase 2 (SOD2), thioredoxin reductase 1 (TXRND1), and heme oxygenase 1 (HO-1) [42]. Likewise, miR-27a was shown to disrupt glutathione- (GSH-) associated antioxidant mechanism by targeting xCT, which mediates the uptake of cystine, a precursor for GSH biosynthesis [36]. miR-324 has been reported to induce ROS level through inhibiting a driver of tumor progression, activated CDC42 kinase 1 (ACK1) [52]. In contrast, miR-373 plays an anti-ROS role in TNBC by targeting thioredoxin-interacting protein (TXNIP), which functions to inhibit antioxidant protein thioredoxin (Trx) by interacting with its redox-specific active cysteine residues [53]. The ROS-suppressing hallmark of miR-373/TXNIP acted to promote HIF-1*α*/twist family BHLH transcription factor 1 (TWIST1) pathway, leading to increased invasive behaviour of TNBC cells [53].

Furthermore, some mitomiRs induce ROS production by targeting genes involved in the integrity of mitochondria. In this regard, miR-4485 was demonstrated to translocate to mitochondria to exert direct binding activity to mtDNA-encoded 16S rRNA, resulting in a decrease in electron transport chain (ETC) enzyme activity and mitochondrial ATP production, as well as an increase in expression level of ROS [44]. The tumor-suppressive effect in vivo was verified by overexpression of miR-4485 in TNBC cells [44]. In addition, Ahir et al. recently identified that miR-34a is implicated in effectively suppressing growth and mammosphere formation of TNBC [54]. The inhibitory role of miR-34a on B cell lymphoma 2 (BCL-2) permits the formation of permeabilization pore on the outer membrane of the mitochondria (OMM), promoting the intracellular ROS production [54].  Table 2 summarizes the mitomiRs acting toward the regulation of redox homeostasis and the biological roles and mechanisms in TNBC.

**Figure 3:**
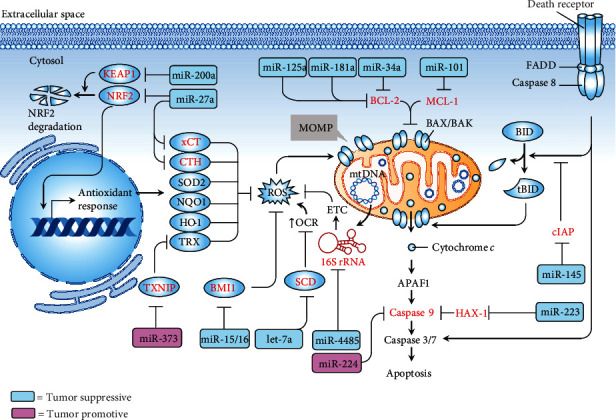
mitomiRs implicated in the regulatory network of redox homeostasis and the fate of apoptosis in the scenario of TNBC. Tumor-suppressive (blue rectangle) or tumor-promotive (pink rectangle) mitomiRs and their target genes (red) act on specific target genes (red). APAF1: apoptotic protease activating factor 1; BAX: Bcl-2-associated X protein; BCL-2: B cell lymphoma 2; BID: BH3 interacting-domain death agonist; BMI1: B cell-specific Moloney murine leukemia virus integration site 1; CTH: cystathionine gamma-lyase; cIAP: cellular inhibitor of apoptosis; ETC: electron transport chain; FADD: Fas-associated protein with death domain; HAX-1: HCLS1-associated protein X-1; HO-1: heme oxygenase 1; MCL-1: induced myeloid leukemia cell differentiation protein Mcl-1; MOMP: mitochondrial outer membrane permeabilization; mtDNA: mitochondrial DNA; NQO1: NAD(P)H quinone dehydrogenase 1; NRF2: nuclear factor erythroid 2-related factor 2; OCR: oxygen consumption rate; SCD: stearoyl-CoA desaturase; ROS: reactive oxygen species; SOD2: superoxide dismutase mitochondrial; tBID: truncated BH3 interacting-domain death agonist; TRAIL: tumor necrosis factor-related apoptosis-inducing ligand; TRX: thioredoxin; TWIST1: twist family BHLH transcription factor 1; TXNIP: thioredoxin-interacting protein; TXNRD1: thioredoxin reductase 1; xCT (encoded by SLC7A11): cystine/glutamate transporter.

Several lines of study have revealed a group of mitomiRs modulate the carcinogenesis and sensitivity to drugs of TNBC by targeting genes implicated in apoptotic signaling. Liu et al. have demonstrated that miR-101 play a positive role in the induction of TNBC apoptosis by targeting induced myeloid leukemia cell differentiation protein Mcl-1 (MCL-1), a prosurvival member of the Bcl-2 family [55]. Overexpression of miR-101 was shown to inhibit tumor growth and sensitize cells to paclitaxel-induced apoptosis [55]. Similarly, Zheng et al. demonstrated that miR-145 has a promotive role in receptor-interacting protein Fas-associated protein with death domain caspase-8 (RIP1-FADD-casp8) apoptosis signaling induced by TNF-*α* [56]. By targeting cellular inhibitor of apoptosis (cIAP1), miR-145 can exert TNBC-suppressive function, leading to the activation of the extrinsic pathway, whereby activated casp8 act to cleave BH3 interacting-domain death agonist (BID) to initiate the mitochondrial apoptotic pathway [56]. miR-233 has been shown to specifically suppress the expression of HCLS1-associated protein X-1 (HAX-1), an antiapoptotic protein that inhibits the activation of caspase-9 [57]. miR-223 overexpression potentiated proapoptotic effect induced by TNF-related apoptosis-inducing ligand (TRAIL) over triple-negative breast cancer stem cells (TNBCSCs) [57]. Patel el al. revealed that miR-15a/miR-16 overexpression in TNBC cells results in increased intrinsic pathway of apoptosis through direct of B cell-specific Moloney murine leukemia virus integration site 1 (BMI1), an oncogene involving mitochondrial homeostasis [58]. miR-20b was shown to target VEGF and cause deceased BCL-2 and increased BAX and activation of cell apoptosis, resulting in an inhibitory effect on proliferation, migration, and invasion of TNBC cells [59]. Both groups, Ahir et al. and Majzoub et al., have recently shown that BCL-2 is a specific target of miR-34a [54] and miR-125a/miR-181a [55]. These mitomiRs can exert suppressive effect on TNBC cells by inducing apoptosis as well as reducing cell migration [55] and tumor growth [54]. Conversely, miR-224 has been reported to be tumor-promotive in TNBC. Zhang et al. showed that miR-224 provides apoptotic resistance and enhances proliferation, migration, and invasion by targeting caspase-9 [60]. Collectively, the recent understandings of mitomiRs in targeting apoptosis pathway in the context of TNBC are listed in Table 3. The schematic sophisticated network encompassing tumor-suppressive/tumor-promotive mitomiRs targeting molecules central to redox homeostasis and apoptosis is illustrated in Figure 3.

## 4. Roles of mitomiRs in Regulating Mitochondrial Dynamics and Mitophagy

Mitochondria are highly dynamic organelles. The dynamic properties comprising fusion, fission, and degradation not only play critical role for their optimal function in energy generation but also have profound impact on human diseases, including cancer [61]. The multiple events of mitochondrial fusion require hydrolysis of GTP and mitochondrial membrane potential [62]. Mitofusins 1 and 2 (MFN1/2) are both GTP-hydrolyzing enzymes of the dynamin superfamily. They are located on the outer membrane of mitochondria (OMM) and required for OMM fusion [63]. The fusion of inner membrane of mitochondria (IMM) is mediated by another member of the dynamin superfamily, optic atrophy 1 (OPA1), located in IMM [64]. On the other hand, the mitochondrial fission largely relied on the activity of dynamin-related protein 1 (DRP1), a GTP-hydrolyzing enzyme [65, 66]. DRP1 is recruited from the cytosol to associate with corresponding receptors residing on OMM, including mitochondrial fission 1 protein (FIS1), mitochondrial fission factor (MFF), mitochondrial dynamics protein MID49 (MID49), and mitochondrial dynamics protein MID51 (MID51) [67–69]. Mitochondrial fission acts to promote segregation of damaged mitochondria, which subsequently facilitates mitochondrial fragments of the appropriate size for engulfment by autophagosomes. The degradation of dysfunctional mitochondria by autophagy, or mitophagy, is a major mechanism for mitochondrial quality control [61].

**Figure 4:**
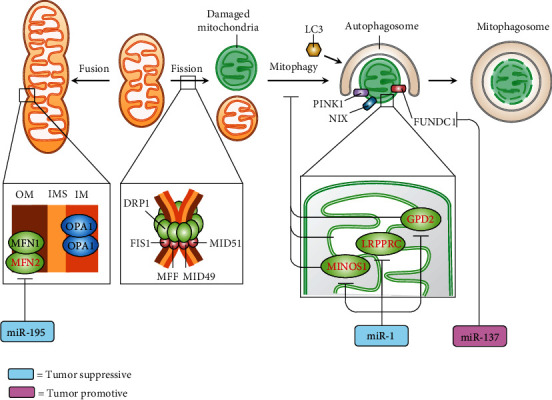
mitomiRs (miR-195, miR-1, and miR-137) regulate mitochondrial quality control machinery, e.g., mitochondrial dynamics and mitophagy. Tumor-suppressive (blue rectangle) or tumor-promotive (pink rectangle) mitomiRs and their target genes (red). DRP1: dynamin-related protein 1; FIS1: mitochondrial fission 1 protein; FUNDC1: FUN14 domain containing 1; GPD2: glycerol-3-phosphate dehydrogenase 2; LC3: microtubule-associated proteins 1A/1B light chain 3B; LDH: lactate dehydrogenase; LRPPRC: leucine-rich pentatricopeptide-repeat containing; MCU: mitochondrial calcium uniporter; MFF: mitochondrial fission factor; MFN: mitofusin; MID49/51: mitochondrial dynamics protein MID49/51; MINOS1: mitochondrial inner membrane organizing system 1; NIX: NIP-3-like protein X; OPA1: optic atrophy 1; PINK1: PTEN-induced kinase 1.

More recently, the emerging role of mitomiRs in TNBC progression via regulating the mitochondrial quality control machinery has been revealed (Figure 4). In this regard, Purohit et al. reported that miR-195 exerts anti-TNBC effect by targeting NFN2. The dysregulated mitochondrial dynamics was evidenced by decreased MFN2 and OPA1, increased DRP1, and enhanced mitochondrial fission. miR-195 also led to declined mitochondrial OXPHOS and bioenergetics and increased ROS level and cell apoptosis [70]. Zhang et al. demonstrated that overexpression of miR-1 exerts a tumor-suppressive effect by simultaneously targeting mitophagy-associated genes including MINOS1 (mitochondrial inner membrane organizing system 1), GPD2 (glycerol-3-phosphate dehydrogenase 2), and LRPPRC (leucine-rich pentatricopeptide-repeat containing), leading to an induction of mitophagy in breast cancer stem cells [71]. On the other hand, Hu et al. showed that miR-137 play a tumor-promotive role in breast cancer stem cells [72]. By direct targeting FUN14 domain containing 1 (FUNDC1), miR-137 repressed the mitophagy by reducing the expression of FUNDC1, NIP-3-like protein X (NIX), and LC3-II, resulting in an increase in mitochondrial biogenesis and bioenergetics and a decrease in ROS level and cell apoptosis [72].  Table 4 highlights the mitomiRs involved in mitochondrial dynamic and mitophagy.

## 5. mitomiRs and Calcium Transport

**Figure 5:**
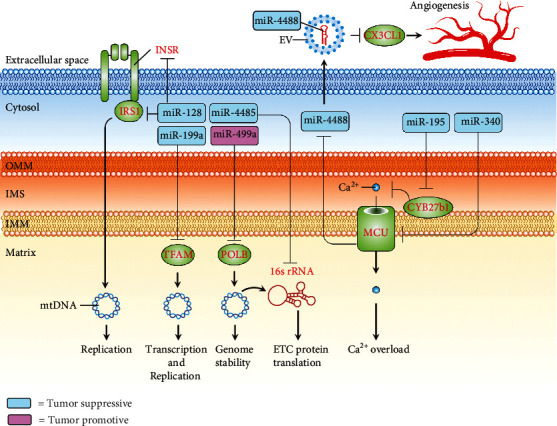
Schematic pipeline depicting mitomiRs that exert tumor-suppressive or tumor-promotive role on TNBC through acting on mechanisms involved in mtDNA maintenance or mitochondrial calcium uptake. Tumor-suppressive (blue rectangle) or tumor-promotive (pink rectangle) mitomiRs and their target genes (red). CX3CL1: C-X3-C motif chemokine ligand 1; CYP27B1: cytochrome P450 family 27, subfamily B, polypeptide 1; INSR: insulin receptor; IRS1: insulin receptor substrate 1; MCU: mitochondrial calcium uniporter; mtDNA: mitochondrial DNA; POLB: DNA polymerase beta; ROS: reactive oxygen species; TFAM: mitochondrial transcription factor A.

A growing body of literature has revealed the implications of mitochondrial calcium uptake in tumorigenesis [73]. Indeed, some of the studies unravelled the roles of a cluster of mitomiRs in inhibiting TNBC progression by disturbing mitochondrial calcium uptake.  Table 4 outlines the recent findings regarding this issue. Specifically, Singh et al. demonstrated that miR-195 acts toward increased mitochondrial calcium by targeting cytochrome P450 family 27, subfamily B, polypeptide 1 (CYP27B1), leading to dysfunctional mitochondria as evidenced by loss of mitochondrial membrane potential. They confirmed its antitumor role by showing that overexpression of miR-195 impedes EMT, proliferation, migration, and invasion of TNBC cells [43]. Yu et al. showed that miR-340 negatively regulates mitochondrial calcium uniporter (MCU) by direct interacting with MCU 3′UTR. Downregulated MCU was shown to inhibit tumor Warburg effect, leading to a significant decrease in cell migration and invasion *in vitro* and lung metastasis *in vivo* [37]. Interestingly, Zheng el al. revealed that knockdown of MCU induces generation and secretion of miR-4488-containing extracellular vesicles (EVs) from TNBC cells. The miR-4488-containing EVs uptaken by endothelial cells acted to target an angiogenic activator C-X3-C motif chemokine ligand 1 (CX3CL1), resulting in suppressed angiogenic activity. Furthermore, the administration of miR-4488-containing EVs inhibited tumor metastasis and increased survival time of tumor-bearing mice [74]. The schematic pipeline regarding the roles and mechanisms of mitomiRs directing at mitochondrial calcium balance is illustrated in Figure 5.

## 6. mitomiRs and mtDNA Maintenance

A line of research identified a group of mitomiRs functioning to modulate the maintenance of mtDNA in the context of TNBC (Table 4). Sripada et al. showed that miR-4485 suppresses tumor growth and cell proliferation of TNBC by targeting 16S rRNA encoded by mtDNA [44]. Fan et al. demonstrated that miR-199a directly targets mitochondrial transcription factor A (TFAM) and causes a significant decrease in mtDNA content, leading to reduced proliferation and potentiate proapoptotic effect induced by cisplatin [75]. In addition, Xiao et al. reported that miR-128 exerts a mtDNA-reducing effect through targeting INSR/IRS1, resulting in a decrease in mitochondrial bioenergetics, proliferation, and tumor growth of TNBC [33]. More recently, miR-499a was identified as a driver of TNBCSC development. In this regard, Manda et al. showed that miR-499a acts to mediate the suppression of DNA polymerase beta (POLB), which plays a master role in the repair and genome stability of mtDNA [76]. We summarized the mechanisms of mitomiRs acting on mtDNA maintenance of TNBC in Figure 5.

## 7. Prognostic Value, Therapeutic Implications, and Future Perspective

The expression of several mitomiRs can potentially be regarded as predictive, diagnostic, and prognostic biomarkers for TNBC (Table 5). A cluster of low-expressed mitomiRs is identified in tumor tissue [28, 33, 34, 44–47, 52, 55, 71, 77–79], while some of them are high-expressed [32, 53, 79, 80]. Of note, some mitomiRs showed clinical significance in predicting disease outcome. There are a group of mitomiRs enabling predicting survival time [33, 34, 45, 47, 77], while another set of them indicates advanced stage or metastasis [52, 53, 55]. Importantly, circulating mitomiRs identified as relevant biomarkers provide an opportunity to establish a rapid clinical panel which can be less invasive and more accessible [47, 48, 74, 79–81].

Recent insights into therapeutic approaches in preclinical model pave new paths to potential treatments for TNBC (Table 6). These strategies exert treatment effect by targeting various aspects implicated in mitochondrial homeostasis, including metabolic reprogramming, apoptosis, redox signaling, and mitochondrial calcium transport. Most common approaches employed genetic manipulation, including siRNA, shRNA, and antisense for mitomiR inhibition [28, 52, 59, 80]. Although some approaches conducted by virus-based gene delivery demonstrated treatment efficiency [33, 82], the potential adverse effects to humans must be taken into consideration in the future should they be registered for clinical trials. The use of chemotherapeutics combo could be promising but still need more advanced study, such as in vivo model (31541355). Nanoparticle-loaded mitomiRs provide a potential way to achieve highly efficient delivery into tumor (32319481). Interestingly, Ahir et al. demonstrated a mitomiR-promoting nanotechnology using copper oxide nanowire fabricated with folic acid (CuO-Nw-FA) that enables enhanced cellular uptake in TNBC cells without imparting significant toxicity in normal cellular system [83]. Oral hypoglycemic drug [48], natural products [76, 84], epigenetic modifier [51], and physical agent [85] have been shown to effectively inhibit TNBC cell proliferation. More notably, the notion of using mitomiR-containing EVs may offer a treatment strategy with advantage of high purity, specificity, and biodistribution as less safety concern [74]. Of note, some clinical trials utilizing mitomiR-based treatment for several types of cancer have been registered (Table 7). Unfortunately, due to the occurrence of immune-related serious adverse events, the trial using miR-34a was terminated (NCT01829971). The termination of anti-miR-155-based drug is due to business reasons, but not concerns regarding safety or lack of efficacy (NCT03713320). The miR-26-based approach, TargomiRs, may be a next glimmer of hope for cancer treatment. The first-in-human, phase 1 trial of TargomiR patients with malignant pleural mesothelioma has completed. The results showed acceptable safety profile and early signs of activity of TargomiRs in patients [86], supporting the feasibility of further study.

## 8. Conclusions

Due to the absence of unambiguous molecular targets for TNBC, its clinical treatment is still a challenging issue. The hallmarks of altered metabolism and mitochondrial fitness in TNBC provide an opportunity for advancing new diagnostic/prognostic tools and therapeutic approaches. mitomiRs are a subgroup of microRNAs that closely regulate mitochondrial functionality by targeting genes present in the cytosol or mitochondria. Growing evidence has elucidated its effectiveness and mechanism of action in the context of TNBC, making mitomiRs an emerging field of study. Indeed, the predictive, prognostic, and diagnostic value of a number of mitomiRs has been revealed. There is increasing novel mitomiR-based therapeutics aiming for efficient inhibition on tumor growth. It is important to note that some mitomiRs were qualified for the early stage of clinical trial. In spite of these inspiring advances, there is still a need to gain deeper insight into mitomiR-dictated mechanisms with respect to mitochondrial homeostasis, to develop accessible prediction panel with satisfactory sensitivity/specificity and to explore interventions suitable for clinical use. To summarize, mitomiRs represent attractive therapeutic targets for the treatment of TNBC.

## Figures and Tables

**Table 1:** Roles and mechanisms of mitomiRs in metabolic reprogramming.

mitomiR	Function	Target gene	Mechanism of action	Outcome	PMID	Reference
*Glucose metabolism*						
miR-29a	(-)TNBC	*ARG2*	↓AGR2→↓HIF-1*α*	↑Apoptosis ↓Proliferation ↓Migration ↓Invasion ↓Tumor growth	33223849	Wang et al., 2020 [28]
miR-210	(+)TNBC	*GPD1L*, *CYGB*	↓GPD1L→↑HIF-1*α*→↑glycolysis ↓CYGB→↓p53→↑glycolysis	↓Apoptosis ↑Tumor growth	32908121	Du et al., 2020 [29]
miR-140	(-)TNBC	*GLUT1*	↓GLUT1→↓glycolysis	↑OCR/↓ECAR ↓Proliferation ↓Tumor growth	31184216	He et al., 2019 [30]
let-7a	(-)TNBC	*STAT3*	↓STAT3→↓hnRNPA1→↓PKM2 →↓Glycolysis	↓Proliferation	30368881	Yao et al., 2019 [31]
miR-155	(+)TNBC	*PIK3R1*, *FOXO3a*	↓PI3K/↓FOXO3a→↑cMYC →↑HK2/↑PKM2/↑LDHA →↑Glycolysis	↑Tumor growth	29527004	Kim et al., 2018 [32]
miR-128	(-)TNBC	*INSR*, *IRS1*	↓INSR/↓INS1→↓p-AKT/↓HK2/↓PFK →↓Glycolysis	↓Mitochondrial bioenergetics ↓Proliferation ↓Tumor growth	29116653	Xiao et al., 2018 [33]
miR-342	(-)TNBC	*MCT1*	↓MCT1→↓lactate uptake	↓Proliferation ↓Migration	30115973	Romero-Cordoba et al., 2018 [34]
miR-30a	(-)TNBC	*LDHA*	↓LDHA→↓glycolysis	↑OCR/↓ECAR ↓Proliferation ↓Migration ↓Invasion ↓Tumor growth ↓Tumor metastasis	28461244	Li et al., 2017 [35]
miR-21	(+)TNBC	*CAB39L*, *SESN1*	↓CAB39L, ↓SESN1→↓p-AMPK→↑mTOR	↑Proliferation ↑Migration ↑Invasion ↑Tumor growth	28698800	Pulito et al., 2017 [36]
miR-340	(-)TNBC	*MCU*	↓MCU→↓[Ca^2+^]_m_→↓LDH →↓Glycolysis	↓ATP production ↓Lactate production ↓Migration ↓Tumor metastasis	29137386	Yu et al., 2017 [37]
miR-101	(-)TNBC	*AMPK*	↓AMPK→↓glycolysis	↓Proliferation	27145268	Liu et al., 2016 [38]
miR-18a	(-)TNBC	*HIF1A*	↓HIF-1*α*→↓hypoxic gene expression	↓Tumor growth ↓Tumor metastasis	25069832	Krutilina et al., 2014 [39]
miR-143	(-)TNBC	*HK2*	↓HK2→↓glycolysis	↑Apoptosis ↓Proliferation ↓Tumor growth	22354042	Jiang et al., 2012 [40]
miR-155	(+)TNBC	*mir-143*	↑miR-155→↓C/EBP*β*→ ↓miR-143→↑HK2→↑glycolysis	↑Tumor growth	22354042	Jiang et al., 2012 [40]
*Fatty acid metabolism*						
miR-1291	(-)TNBC	*ESRRA*	↓ERR*α*→↓CPT1C	↓Proliferation ↓Tumor growth ↑TNBC sensitivity to 2-DG	32641987	Chen et al., 2020 [41]
let-7a	(-)TNBC	*SCD*	↓SCD, ↓G6PD, ↓FASN, ↓ASSDHPPT →↑OCR/↑ECR/↑*ΔΨ*m	↓Proliferation ↑TNBC sensitivity to doxorubicin	25669981	Serguienko et al., 2015 [42]
miR-195	(-)TNBC	*ACACA*, *FASN*, *HMGCR*	↓Cellular triglyceride ↓Cellular cholesterol ↓*ΔΨ*m	↓EMT ↓Proliferation ↓Migration ↓Invasion	26632252	Singh et al., 2015 [43]
*Glutamine/cystine metabolism*						
miR-27a	(-)TNBC	*SLC7A11*, *CTH*	↓xCT→↓cystine uptake ↓CTH→↓cystine production	↓Mammosphere formation ↓CSC markers ↑TNBC sensitivity to doxorubicin and paclitaxel	32066826	Ueda et al., 2020 [36]
*OXPHOS*						
miR-4485	(-)TNBC	*16S rRNA*	↓16S rRNA→↓ETC enzymes	↑Cell death ↓Proliferation ↓Tumor growth	28220193	Sripada et al., 2017 [44]
miR-663	(-)TNBC	*UQCC2*	↓UQCC2→↓complex III activity	↑OXPHOS subunits ↓Invasion ↓Tumor growth	29066618	Carden et al., 2017 [45]

(+): oncogenic; (-): tumor suppressor; “↑”: enhanced; “↓”: reduced; *ΔΨ*m: mitochondrial membrane potential; AASDHPPT: 4-phosphopantetheinyl transferase; ACACA: acetyl-CoA carboxylase; AGR2: anterior gradient 2; [Ca^2+^]_m_: mitochondrial calcium; AMPK: AMP-activated protein kinase; CAB39L: calcium-binding protein 39-like; C/EBP*β*: CCAAT-enhancer-binding protein *β*; CPT1C: carnitine palmitoyltransferase 1C; CTH: cystathionine gamma-lyase; CYGB: cytoglobin; ECAR: extracellular acidification rate; EMT: epithelial-mesenchymal transition; ERR*α* (encoded by *ESRRA*): estrogen-related receptor alpha; FASN: fatty acid synthase; FOXO3a: forkhead box O3; G6PD: glucose-6-phosphate dehydrogenase; GLUT1: glucose transporter 1; GPD1L: glycerol-3-phosphate dehydrogenase 1; *HIF1A*/HIF-1*α*: hypoxia-inducible factor 1-alpha; HK2: hexokinase 2; HMGCR: 3-hydroxy-3-methylglutaryl CoA reductase; INSR: insulin receptor; IRS1: insulin receptor substrate 1; LDHA: lactate dehydrogenase A; MCT1: monocarboxylate transporter 1; MCU: mitochondrial calcium uniporter; mtDNA: mitochondrial DNA; mt-ROS: mitochondrial reactive oxygen species; OCR: oxygen consumption rate; OXPHOS: oxidative phosphorylation; PFK: phosphofructokinase; PI3K: phosphoinositide 3-kinases; PKM2: pyruvate kinase M1/2; SCD: stearoyl-CoA desaturase; SESN1: sestrin-1; STAT3: signal transducers and activators of transcription 3; UQCC2: ubiquinol-cytochrome c reductase complex assembly factor 2; xCT (encoded by *SLC7A11*): cystine/glutamate transporter.

**Table 2:** Roles and mechanisms of mitomiRs in the perturbation of redox homeostasis.

mitomiR	Function	Target gene	Mechanism of action	Outcome	PMID	Reference
miR-34a	(-)TNBC	*BCL-2*	↓BCL-2/↑BAX →↑ROS/↓*ΔΨ*m	↓Mammosphere formation ↓Tumor growth	32319481	Ahir et al., 2020 [54]
miR-27a	(-)TNBC	*SLC7A11* *CTH* *NFE2L2*	↓xCT, ↓CTH, ↓NRF2 →↑ROS, ↓autophagic flux	↓Mammosphere formation ↓CSC markers ↑TNBC sensitivity to doxorubicin and paclitaxel	32066826	Ueda et al., 2020 [36]
miR-324	(-)TNBC	*ACK1*	↓ACK1→↑ROS→ ↑DNA oxidative damage	↓Proliferation ↓Tumor growth	31751910	Zhang et al., 2019 [52]
miR-4485	(-)TNBC	*16S rRNA*	↓16S rRNA→↓ETC enzyme activity→↑ROS/↓*ΔΨ*m	↑Cell death ↓Proliferation ↓Tumor growth	28220193	Sripada et al., 2017 [44]
let-7a	(-)TNBC	*SCD*	↓SCD→↑OCR→↑ROS	↑SOD2, TXNRD1, HO-1 ↓Proliferation ↑Cell cycle arrest ↑TNBC sensitivity to doxorubicin	25669981	Serguienko et al., 2015 [42]
miR-373	(+)TNBC	*TXNIP*	↓TXNIP→↑TRX→↓ROS→↑HIF-1*α*→↑TWIST 1→↑miR-373	↑EMT ↑Migration and invasion ↑Tumor growth ↑Tumor metastasis	26196741	Chen et al., 2015 [53]
miR-200a	(-)TNBC	*KEAP1*	↓KEAP1→nuclear translocation of NRF2→↑NQO1	↑Antioxidant pathway ↓Anchorage-independent cell growth	21926171	Eades et al., 2011 [51]

(+): oncogenic; (-): tumor suppressor; “↑”: enhanced; “↓”: reduced; *ΔΨ*m: mitochondrial membrane potential; ACK1: activated CDC42 kinase 1; BAX: Bcl-2-associated X protein; BCL-2: B cell lymphoma 2; CTH: cystathionine gamma-lyase; CSCs: cancer stem cells; ETC: electron transport chain; HAX-1: HCLS1-associated protein X-1; HO-1: heme oxygenase 1; NQO1: NAD(P)H quinone dehydrogenase 1; NRF2 (encoded by *NFE2L2*): nuclear factor erythroid 2-related factor 2; ROS: reactive oxygen species; SCD: stearoyl-CoA desaturase; SOD2: superoxide dismutase mitochondrial; TRAIL: tumor necrosis factor-related apoptosis-inducing ligand; TNBCSC: triple-negative breast cancer stem cell; TRX: thioredoxin; TWIST1: twist family BHLH transcription factor 1; TXNIP: thioredoxin-interacting protein; TXNRD1: thioredoxin reductase 1; xCT (encoded by *SLC7A11*): cystine/glutamate transporter.

**Table 3:** Roles and mechanisms of mitomiRs in cell apoptosis.

mitomiR	Function	Target gene	Mechanism of action	Effect on TNBC	PMID	Reference
miR-34a	(-)TNBC	BCL2	↓BCL-2→↑BAX	↑Apoptosis ↓Migration ↓Mammosphere formation ↓Tumor growth	32319481	Ahir et al., 2020 [54]
miR-224	(+)TNBC	CASP9	↓Caspase-9	↓Apoptosis ↑Proliferation ↑Migration ↑Invasion	30886656	Zhang et al., 2019 [60]
miR-125a/miR-181a	(-)TNBC	BCL2	↓BCL-2→↑BAX	↑Phospho-p53 ↑PARP cleavage ↑Apoptosis	31541355	Majzoub et al., 2019
miR-20b	(-)TNBC	VEGF	↓BCL-2→↑BAX →Caspase-9/3	↑Apoptosis ↓Proliferation ↓Migration ↓Invasion	28550685	Lu et al., 2018 [59]
miR-15/16	(-)TNBC	BMI1	↓BCL-2/↑BAX/↑BID →↓*ΔΨ*m→↑caspase-9/3	↑Cell death ↓Proliferation ↓Migration	27596816	Patel et al., 2016 [58]
miR-223	(-)TNBC	HAX1	↓HAX-1	↑TRAIL-induced apoptosis of TNBCSC	27618431	Sun et al., 2016 [57]
miR-145	(-)TNBC	cIAP	↓cIAP→↑FADD→ ↑Caspase-8/3→↑tBID	↑TNF-*α*-induced apoptosis	26733177	Zheng et al., 2016 [56]
miR-101	(-)TNBC	MCL1	↓MCL-1→↓*ΔΨ*m	↑Apoptosis ↓Proliferation ↓Tumor growth ↑TNBC sensitivity to paclitaxel	26036638	Liu et al., 2015 [55]

2-DG: 2-deoxy-D-glucose; *ΔΨ*m: mitochondrial membrane potential; BAX: Bcl-2-associated X protein; BCL-2: B cell lymphoma 2; BID: BH3 interacting-domain death agonist; CASP9: caspase-9; cIAP: cellular inhibitor of apoptosis; FADD: Fas-associated protein with death domain; HAX-1: HCLS1-associated protein X-1; MCL-1: induced myeloid leukemia cell differentiation protein Mcl-1; PARP: poly ADP-ribose polymerase; tBID: truncated BH3 interacting-domain death agonist; TRAIL: tumor necrosis factor-related apoptosis-inducing ligand; TNBCSC: triple-negative breast cancer stem cell; VEGF: vascular endothelial growth factor.

**Table 4:** Roles and mechanisms of mitomiRs in mitochondrial homeostasis on dynamics, mitophagy, calcium transport, and mtDNA maintenance.

mitomiR	Function	Target gene	Mechanism of action	Outcome	PMID	Reference
*Mitochondrial dynamics*						
miR-195	(-)TNBC	*MFN2*	↓MFN2 ↓OPA1 ↑DRP1	↑Mitochondrial fission ↓OCR ↓Mitochondrial ATP ↑ROS ↑Apoptosis	30932749	Purohit et al., 2019 [70]
*Mitophagy*						
miR-137	(+)BCSC	*FUNDC1*	↓FUNDC1/↓NIX/↓LC3-II →↓Mitophagy	↑Mitochondrial biogenesis ↓ROS ↑ATP production ↓Apoptosis	32945512	Hu et al., 2020 [72]
miR-1	(-)BCSC	*MINOS1* *GPD2* LRPPRC	↓MINOS1/↓GPD2/↓LRPPRC →↑Disorganized cristae →↑PINK1→↑LC3-II →↑Mitophagy	Cell cycle arrest at G0/G1 ↓Tumor growth	31765945	Zhang et al., 2019 [71]
*Mitochondrial calcium transport*						
miR-4488	(-)TNBC	*CX3CL1*	↓MCU (TNBC)→ ↑miR-4488 EVs (TNBC)→ ↓CX3CL1 (ECs)	↓Angiogenesis ↓Tumor metastasis ↑Survival time of mice	33067576	Zheng et al., 2020 [74]
miR-340	(-)TNBC	*MCU*	↓MCU→↓[Ca^2+^]_m_ →↓LDH	↓Glucose uptake ↓ATP production ↓Lactate production ↓Migration ↓Tumor metastasis	29137386	Yu et al., 2017 [37]
miR-195	(-)TNBC	*CYP27b1*	↑[Ca^2+^]_m_ ↑[Ca^2+^]_C_ ↓*ΔΨ*m	↓EMT ↓Proliferation ↓Migration ↓Invasion	26632252	Singh et al., 2015 [43]
*mtDNA maintenance*						
miR-499a	(+)TNBCSC	*POLB*	↑POLB→↑mtDNA stability	↑Tumorigenic genes ↑CSC genes ↑Proliferation	33278391	Manda et al., 2020 [76]
miR-128	(-)TNBC	*INSR*, *IRS1*	↓INSR/↓INS1→↓mtDNA content	↓Mitochondrial bioenergetics ↓Proliferation ↓Tumor growth	29116653	Xiao et al., 2018 [33]
miR-199a	(-)TNBC	*TFAM*	↓TFAM→↓mtDNA content	↑Cisplatin-induced apoptosis ↓Proliferation	28126676	Fan et al., 2017 [75]
miR-4485	(-)TNBC	*16S rRNA*	↓16S rRNA→↓ETC enzyme activity→↓mitochondrial ATP	↑Cell death ↓Proliferation ↓Tumor growth	28220193	Sripada et al., 2017 [44]

(+): oncogenic; (-): tumor suppressor; “↑”: enhanced; “↓”: reduced; *ΔΨ*m: mitochondrial membrane potential; BCSC: breast cancer stem cell; ↑[Ca^2+^]_C_: cytosolic calcium; [Ca^2+^]_m_: mitochondrial calcium; CX3CL1: C-X3-C motif chemokine ligand 1; CYP27B1: cytochrome P450 family 27, subfamily B, polypeptide 1; DRP1: dynamin-related protein 1; ECs: endothelial cells; EVs: extracellular vesicles; FUNDC1: FUN14 domain containing 1; GPD2: glycerol-3-phosphate dehydrogenase 2; INSR: insulin receptor; IRS1: insulin receptor substrate 1; LC3: microtubule-associated proteins 1A/1B light chain 3B; LDH: lactate dehydrogenase; LRPPRC: leucine-rich pentatricopeptide-repeat containing; MCU: mitochondrial calcium uniporter; MFN: mitofusin; MINOS1: mitochondrial inner membrane organizing system 1; NIX: NIP-3-like protein X; OPA1: optic atrophy 1; PINK1: PTEN-induced kinase 1; POLB: DNA polymerase beta; ROS: reactive oxygen species; TFAM: mitochondrial transcription factor A; UQCC2: ubiquinol-cytochrome C reductase core protein 2.

**Table 5:** Clinicopathological relevance of mitomiRs.

mitomiRs	Specimen	Expression	Clinical relevance	PMID	Reference
miR-4488	Serum EVs	↓	Low expression in TNBC patients	33067576	Zheng et al., 2020 [74]
miR-21	Serum	↓	Decreased level in BC patients after metformin treatment	28698800	Pulito et al., 2017 [48]
miR-34a	Serum Tissue	↓	Low expression predicts poor OS of TNBC patients	31616515 31897330	Li et al., 2019 [47] Kim et al., 2019 [77]
miR-29a	Tissue	↓	Low expression predicts poor OS of BC patients	33223849	Wang et al., 2020 [28]
miR-1	Tissue	↓	Low expression in BC patients	31765945	Zhang et al., 2019 [71]
miR-324	Tissue	↓	Low expression correlated with large tumor size and advanced TNM stage (III-IV) of BC patients	31751910	Zhang et al., 2019 [52]
miR-342	Tissue	↓	Low expression predicts poor OS of TNBC patients	30115973	Romero-Cordoba et al., 2018 [34]
miR-128	Tissue	↓	Low expression predicts poor OS and DFS of TNBC patients	29116653	Xiao et al., 2018 [33]
miR-125b	Tissue	↓	Low expression in BC tissue and TNBC cell line	29434858	Hu et al., 2018
miR-4485	Tissue	↓	Low expression in BC patients	28220193	Sripada et al., 2017 [44]
miR-663	Tissue	↓	Low expression predicts poor OS of BC patients	29066618	Carden et al., 2017 [45]
miR-195	Tissue	↓	Low expression in TNBC patients	29183284	Qattan et al., 2017 [79]
miR-148a, miR-152	Tissue	↓	Low expression in TNBC tissue compared to adjacent normal tissue	25703326	Xu et al., 2015 [46]
miR-101	Tissue	↓	Low expression associated with advanced TNM stage (III-IV) and lymph node infiltration of TNBC patients	26036638	Liu et al., 2015 [55]
miR-195	Plasma	↑	High expression in TNBC patients	29183284	Qattan et al., 2017 [79]
miR-210	Plasma	↑	High expression in TNBC patients	29183284	Qattan et al., 2017 [79]
miR-16	Plasma	↑	High expression in TNBC patients	29183284	Qattan et al., 2017 [79]
miR-221	Serum Tissue	↑	High expression in serum and tumor tissue of BC patients	26503209	Ye et al., 2016 [80]
miR-27a	Serum	↑	High expression in BC patients and in TNBC cell line	26662313	Zhou et al., 2016 [81]
miR-155	Tissue	↑	High expression in tumor tissue indicates elevated glucose uptake of TNBC patients	29527004	Kim et al., 2018 [32]
miR-210	Tissue	↑	High expression in TNBC patients	29183284	Qattan et al., 2017 [79]
miR-16	Tissue	↑	High expression in TNBC patients	29183284	Qattan et al., 2017 [79]
miR-373	Tissue	↑	High expression predicts lymph node metastasis of BC patients	26196741	Chen et al., 2015 [53]

“↑”: enhanced; “↓”: reduced; BC: breast cancer; DFS: disease-free survival; EVs: extracellular vesicles; OS: overall survival; TNBC: triple-negative breast cancer; TNM: tumor, node, metastasis.

**Table 6:** mitomiR-based therapeutics and targeting mechanisms in preclinical study of TNBC.

Approach/reagent	Effecting mitomiR	Experimental model	PMID	Reference
*Metabolic reprogramming*				
siRNA-circPVT1	↑miR-29a	*In vivo*, *in vitro*	33223849	Wang et al., 2020 [28]
Lentivirus-miR-128	↑miR-128	*In vivo*, *in vitro*	29116653	Xiao et al., 2018 [33]
Small molecule (metformin)	↓miR-21	*In vitro*, *in vivo*	28698800	Pulito et al., 2017 [48]
*Apoptosis*				
Nanoparticle-loaded miR-34a	↑miR-34a	*In vivo*, *ex ovo*, *in vitro*	32319481	Ahir et al., 2020 [54]
Nanoparticle-loaded anti-miR-10b	↓miR-10b	*In vivo*, *ex ovo*, *in vitro*	32319481	Ahir et al., 2020 [54]
Cisplatin+thiosemicarbazone compound 4	↑miR-125a, ↑miR-181a	*In vitro*	31541355	Majzoub et al., 2019
shRNA-lncCAMTA1	↑miR-20b	*In vitro*	28550685	Lu et al., 2018 [59]
Anti-miR-221	↓miR-221	*In vitro*	26503209	Ye et al., 2016 [80]
*Redox signaling*				
Natural product (ursolic acid)	↓miR-499a	*Ex ovo*, *in vitro*	33278391	Manda et al., 2020 [76]
Physical agent (ultrasound)	↑miR-200c	*In vitro*	32313093	Shi et al., 2020 [85]
Natural product (parthenolide)	↑miR-29b	*In vitro*	31313842	De Blasio, 2019 [84]
shRNA-LINC00963	↑miR-324	*In vitro*, *in vivo*	31751910	Zhang et al., 2019 [52]
Adenovirus-MDA-7	↓miR-221	*In vitro*, *in vivo*	30842276	Pradhan et al., 2019 [82]
CuO-Nw-FA	↑miR-425	*In vivo*, *ex ovo*, *in vitro*	26520043	Ahir et al., 2016 [83]
Histone deacetylase inhibitor (SAHA)	↑miR-200a	*In vitro*	21926171	Eades et al., 2011 [51]
*Mitochondrial calcium transport*				
Exosome	↑miR-4488	*In vivo*, *in vitro*	33067576	Zheng et al., 2020 [74]

“↑”: enhanced; “↓”: reduced; circPVT1: circular RNA PVT1; CuO-Nw-FA: copper oxide nanowire conjugated with folic acid; lncRNA: long noncoding RNA; MITF: melanogenesis-associated transcription factor; SAHA: suberoylanilide hydroxamic acid.

**Table 7:** Interventional clinical trials for mitomiRs.

Intervention	Effecting mitomiR	Cancer type	Trial number	Phase (status)
*Metabolic reprogramming*				
Cobomarsen (MRG-106)	↓miR-155	T-cell lymphoma/mycosis fungoides	NCT03713320; NCT03837457	Phase 2 (terminated)
TargomiRs	↑miR-16	Mesothelioma NSCLC	NCT02369198	Phase 1 (completed)
*Apoptosis*				
MRX-34	↑miR-34a	Liver cancer	NCT01829971	Phase 1 (terminated)
		Lymphoma		
		SCLC		
		NSCLC		
		Melanoma		
		Multiple myeloma		
		Renal cell carcinoma		

“↑”: enhanced; “↓”: reduced; NSCLC: non-small-cell lung cancer; SCLC: small cell lung cancer.

## Data Availability

No data were used to support this study.
